# Sanitation Infrastructure at the Systemic Edge: Segregated Roma Settlements and Multiple Health Risks in Slovakia

**DOI:** 10.3390/ijerph18116079

**Published:** 2021-06-04

**Authors:** Richard Filčák, Daniel Škobla

**Affiliations:** 1 Center of Social and Psychological Sciences, The Institute for Forecasting, Slovak Academy of Sciences, 811 05 Bratislava, Slovakia; 2 Institute of Ethnology and Social Anthropology, Slovak Academy of Sciences, 813 64 Bratislava, Slovakia; Daniel.skobla@savba.sk

**Keywords:** sanitation infrastructure, multiple health risks, segregated Roma settlements, local government, Slovakia

## Abstract

This article explores how multiple health risks in municipalities with Roma settlements in Slovakia are related to the varieties of local governance and the authorities’ conduct towards the local Roma population. The first part of the paper describes the situation in Roma settlements from the perspective of unequal access to sewerage and water pipelines. Introduced here are data on identified contagious diseases that correlated multiple health risks with the lack of sanitation and/or water infrastructure. The second section of the paper put forth typologies of government approaches towards the Roma, which based on ethnographic fieldwork, allows us to identify factors of attitudinal, structural and policy-oriented nature. Research results point to a “triad” of key circumstances: these are the structural conditions in municipalities and the history of local inter-ethnic relations and attitude of authorities towards Roma. Finally, possible solutions and approaches regarding how to mitigate the multiple health risks are discussed. It is suggested that on the one hand, in many villages there is a profound institutional discrimination of Roma with respect to water and sanitation infrastructure; on the other hand, water services are increasingly becoming an expensive commodity that not everyone can afford. The article concludes with discussion on enabling conditions and ways to ensure access to basic infrastructure in rural Roma communities. The solution is not only a compliance with principles of non-discrimination and existing technical norms and standards but also in securing the accessible funding for construction of the sanitation infrastructure in a smart way, including innovations and operation of cheaper and environmentally responsible sanitation technologies.

## 1. Introduction

Poor health among Roma in Slovakia living in segregated communities is often incorrectly interpreted as being the result of their “traditional” way of life or, as a consequence, of a “culture of poverty”. Pictures of hundreds of shanty dwellings lacking very basic access to water and sanitation are one of the characteristics of many localities in which poverty-stricken Roma live. Sewerage is often absent and a receptacle such as a pit in the earth for use as a toilet located on slopes above settlements or in close proximity to water sources is not rare. Yet, what are viewed as being the ramifications of a particular mode of living, in fact, are implications of structural barriers that cannot be simply overcome by individuals acquiring proper attitudes or cultural competency.

This article explores how multiple health risks in municipalities with Roma settlements in Slovakia are related to the varieties of local governance and the authorities’ conduct towards the local Roma population. It is argued that the absence of basic physical sanitation infrastructure inevitably leads to higher exposure of inhabitants and this correlation is clearly manifested by the disproportionately higher prevalence of contagious and waterborne diseases among Roma living in settlements in comparison to the general population [1].

According to international comparative surveys, 87% of the Romani population in Slovakia live below the poverty line and are the victims of unequal treatment and victimisation by the majority of the population, authorities and the media [2,3]. There are an estimated 250,000 marginalised Roma (approximately 5% of the total population of the country) who suffer from profound institutional discrimination and face widespread unemployment and poverty [4]. They are often scapegoated and blamed by right-wing populists for general social problems and economic misery [5]. Most of these households that can be characterised as low-income face rampant unemployment are trapped in debt and are dependent on social assistance [6].

Research attention both internationally [7,8,9,10,11,12,13,14,15] and in Slovakia [16,17,18,19,20,21,22,23,24,25] has been focused on the question of basic physical infrastructure in segregated Romani ghettos, neighbourhoods or settlements. Some researchers have previously also focused on identifying barriers in access to potable water and water infrastructure [26]. A report by the European Roma Rights Centre [3] indicates that significant numbers of Roma suffer disproportionately from the failures of public authorities to secure access to water and sanitation. Furthermore, it was proven that Roma, especially those living segregated on the outskirts of municipalities, are often treated discriminatively by local authorities with regard to the provision of public utilities [22]. Institutional discrimination and the lack of compliance with technical and health norms and standards pertain also to waste collection carried out by municipal authorities in Roma settlements [4,5,24].

There is increasing literature and practise regarding measurements and indexes for measuring the sustainability of water and the sewerage service system [27]. From originally very technical perspectives focusing on environmental-technical systems, production costs and volumes, there is an increasing focus on also including into the indexes social aspects, including access to the resources, affordability and community management of the resources. From this perspective we find an inspiring need to study Roma communities’ SSI (sanitation sustainability index) [28] and especially the WASSI (Water and Sanitation Sustainability Index) [29]. WASSI operates with descriptors of availability, infrastructure, equity, access, planning, participation, use, impact and satisfaction. As the authors of the indexes point out, water and sewerage systems need to address key environmental and social issues to enhance the sustainability of the solutions [30,31]. Applying experience and approaches developed by SSI, WASSI and other indexes may provide a valuable tool for decision making and addressing the lack of critical infrastructure in the Roma settlements and it is foreseen in the follow up phase of our research.

While both EU and national legislation enforcing fundamental rights and securing the execution of environmental and health norms apply to everybody, in social practice can be found significant irregularities. Roma settlements are areas to which we can apply the concept of the Systemic Edge [32], since living conditions here take on a format so extreme that it cannot be easily captured and becomes invisible and ungraspable [33]. The Systemic Edge concept elucidates the working of social dynamics that push people out of society. The lack of access to physical sanitation infrastructure in settlements in which Roma live as the phenomenon cannot be understood outside of a deeper understanding of social exclusion processes and the exclusion dynamic of the Systemic Edge. This dynamic is propelled and reinforced by racial prejudices and widespread anti-Gypsyism present in society, which, in turn, are reproducing inequalities along ethnic lines manifested in miserable living conditions of the Roma [5,6,34,35,36].

Recent research [37] identified almost 800 segregated Roma settlements in the territory of Slovakia, with extremely poor living conditions characterised by dilapidated housing and an absence of physical infrastructure such as water pipelines and sewerage. Moreover, more than 300 Roma localities were completely disconnected from a water supply. An absence of sewerage was identified in 450 Roma settlements and approximately one third of these settlements discharge sewage, i.e., a mixture of wastewater and excrement, freely to nearby surroundings. Roma in the settlements are often “pushed out” and getting off the standard governance practices resting on the law and norms, and are gravitating towards zones in which the “normal” or “standard” rules do not apply and extreme poverty of inhabitants is complemented by intense policing and repression [21,22,25]. At the same time, the absence of physical infrastructure in settlements and the non-observance of norms and standards in the sphere of public health on the part of municipal authorities are largely overlooked [4,5].

## 2. Research Questions and Methods

The aim of the research was to explore the conduct of local governments in addressing multiple health risks in Roma settlements, which we considered to be located at the so-called Systemic Edge [32]. The research methodology was based on qualitative fieldwork with the aim of building evidence through an ethnographic approach. The level of analyses constituted the Slovak Republic and its 800 Roma settlements, with an approximate population of 250,000.

The research aspired to answer two interlinked research questions: 1. What are typical municipalities’ conduct and responses in managing multiple health risks regarding the local Roma population? 2. What are the viable policies addressing the multiple health risks in a way which shall enhance social dynamics integrating disadvantaged Roma, in contrast to dynamics excluding those marginalised from society? The assumption was that effective mitigation of multiple health burdens in Roma settlements is closely related to the presence or absence of sanitation infrastructure. In this respect it was also supposed that at the Systemic Edge there were discrepancies between official policies, valid legislation, norms, standards (on the one hand) and social practice (on the other hand).

Preparation for qualitative fieldwork proceeded in several steps. As a springboard for the assignment desk research on sanitation infrastructure as a factor in the mitigation of multiple health risks, comprising specifics of the situations in EU countries and in Slovakia, was conducted. Thereafter, a conceptual framework for understanding access to sanitation as being a human right and in the context of supranational declarations, directives and conventions, was designed. In a further step, in order to inform sampling for the qualitative research, it was decided upon the sequential use of quantitative and qualitative data. To obtain an overall picture and statistical information regarding Roma settlements, sanitation and the public health situation, data published elsewhere were used. These came from publicly available resources: (i) Atlas of Roma Communities 2019, quantitative mapping of access to sanitation and wastewater; (ii) the database of the Office of Public Health Authority on the prevalence of contagious diseases; and (iii) the database of the Ministry of the Environment of the Slovak Republic on water and sewerage.

Based on the list of municipalities with Roma inhabitants and with a prevalence of infectious diseases, a sample for ethnographic fieldwork was selected. The sample for the field visits was divided between villages with small (up to 1000 inhabitants), medium-sized (1000–2000 inhabitants) and large settlements (more than 2000 inhabitants). Villages were located in two historic regions of Slovakia-Šariš and Spiš, which are peripheral, currently economically disadvantaged and characterised by rampant unemployment. Two regions with similar characteristics were chosen in order to obtain a socio-economically, ethnically and culturally relatively homogeneous environment and thus eliminating the effect of exogenous factors (e.g., in economically peripheral regions versus economically core regions). The visit of localities usually started by talking with the mayors or officials. The advantage of individual interviews was the possibility of more focused discussion, which was not conditioned by the power relationship to the other interview participants. Thus, apart from the self-censorship and relationship to the researchers there were no other additional factors influencing the respondents’ perspectives. The interview was conducted in a semi-structured way so that the respondents had ample opportunity to state their own opinions, while at the same time a list of questions ensured that we discussed all important points. The questions oscillated around the sanitation infrastructure and the absence of it, the history and plans for future in this respect, and occurrence of contagious diseases in locality. The questions, however, also touched ethnic stereotypes and attitudes of those in power toward impoverished Roma. Interviews usually last between 30 min and one hour. All the respondents were assured that all actual names of persons will not be mentioned in order to protect their anonymity, in order to respect the confidentiality with which respondents shared their perspectives. Thus, this research was by no means designed as a tool for monitoring the work of municipal officials but as an instrument for scientific inquiry.

In total, semi-structured interviews with local stakeholders such as mayors, municipal administrators, elected municipal representatives, social workers, health authorities and local NGOs were conducted in 22 villages. The strategy to prevent bias regarding the choice of interviewees was to secure complementary perspectives. Thus information from village officials were then compared with the perspective of members from the Roma community. Talks with Roma were often made as group discussions outside their homes, where they hung out with their neighbours, and were likely to be less structured than in the case of interviews with officials. The choice of topic was also, to some extent, subject to information obtained earlier from the officials. In total, it was conducted 90 interviews, which were not recorded but from which notes were taken. At the end of every working day, a team of researchers completed obtained information and conducted coding of data. In the course of the visits an ethnographic observation, which allowed us to observe the real situation in the field, as well as the dynamics of interactions and communication between local authorities and Roma inhabitants, was undertaken. The field research was carried out in January and February 2020 (just before the outbreak of the COVID-19 pandemic in Europe). In addition to the data obtained during the desk research and fieldwork, some of the opinions presented in this article are also based on researchers’ long-term sociological experience in social exclusion and multiple disadvantages of Roma in Slovakia.

## 3. Results

Background information for the research was that of research data [37], which monitors connection to the water networks and its real use, including the utilisation of own or public wells, or of non-standard water sources (e.g., rivers or streams), and methods of discharging sewage water, connection to public sewerage, the use of wastewater treatment plants, cesspits or the absence of any of the aforementioned. A significant number of settlements do not possess basic physical infrastructure. While 76% of Roma households located on the outskirts of villages have potential access to water pipelines, only 59% of households are actually connected to pipelines and use them. With regard to sewerage systems, a total of 51% of Roma households potentially have access to public sewerage, but only 35% are actually connected to and use it.

A further important source of information was a list of municipalities with identified contagious diseases from our previous research [1] that correlated multiple health risks with the absence of sanitation and/or water infrastructure. Diseases that were considered were those with transfer typically taking place via the so-called faecal–oral route, where there is a strong element of inadequate hygiene of hands infected (Hepatitis A, Hepatitis E, Rotavirus enteritis, Campylobacterial enteritis, Shigella flexneri and Shigella sonnei). According to this list, during the years 2014–2018 there were 4693 cases of outbreaks of the aforementioned diseases recorded (which occurred in 681 municipalities). Eighty-five per cent of the cases of disease outbreaks appeared in municipalities in which at least a proportion of the Roma residents do not have access to water pipelines and sewerage systems, and another 9% of the cases occurred in municipalities in which all Roma households are without connection to a sewerage system. In contrast, only 15% of the diseases occurred in municipalities in which Roma households were connected to a water supply and sewerage system [1].

The absence of basic infrastructure or barriers in access to infrastructure in Roma settlements, with consequent multiple occurrences of outbreaks of contagious diseases, may have multiple reasons and local varieties; however, our research results point to a “triad” of key factors. These are the structural conditions in municipalities, the history of local inter-ethnic relations and attitudes of authorities towards Roma. The following typologies, which are rather ideal types of local governmentality are based on detailed data, consisting of the mix of (i) structural variables (e.g., sanitation, demography, presence of development activities, presence of health risks-contagious diseases) and (ii) fieldwork data from interviews and observations. Table 1 shows the research grid, which shows clearly how it was proceeded to obtain the results. For each visited village, type of respondents, number of individual interviews, number of group discussions and number of coded information from fieldwork notes, in order to allow comparisons an analysis, is clearly indicated in the table. In correlation with the set of structural variables for each locality the research results were as follows. This section thus is synthesis of data based on nuanced, detailed qualitative analysis.

A type: Rural slums in inert and economically disadvantaged municipalities. This type represents villages with densely populated Roma settlements, consisting mostly of closely packed, decrepit shacks, with deteriorating or absent physical infrastructure. In this type of village, we see Roma inhabitants living in one or even more segregated settlements. Settlements either are completely without a regular source of water and sewerage or depend on a limited number of water pumps and/or wells whose quality often does not comply with hygienic norms and regulations and is insufficient when considering the size of the settlement. The number of the Roma population in a settlement may exceed the number of the non-Roma population in the village. Municipalities typically do not possess any land for housing development, and the land under Roma dwellings is owned by non-Roma proprietors and is partitioned into small lots as a result of an inheritance process (in the post-communist restitutions in the 1990s). Reaching an agreement with owners regarding buying such lots is usually costly (and for smaller villages with low budget unaffordable) and extremely difficult and requires extensive negotiations between municipality authorities and owners. Typically, sanitation infrastructure here ends on the verge of village, is not extended to the settlement and is the under the ownership (or long-term lease) of the private or state-owned water company, which devoid local authorities over decisions and the prices. The mayor in this type of village may or may not be of a Roma background but he/she and municipal representatives may relatively positively lean towards local Roma and be open to addressing problems such as inadequate housing, economic impoverishment, and public health. It is the economic deprivation of the municipality, the absence of municipality-owned land, and often also the lack of political “connections” of the mayor, expert knowhow and human capacities which impede tackling the multiple health risks.

B type: Problem-oriented municipalities on the developmental path. This type represents larger villages with a large Roma population, which, due to a combination of a better overall economic situation of households and relatively better living conditions and a record of some developmental activities or projects implemented in a village, make gradual progress. Structurally, these villages, including their Roma settlements on the outskirts, are equipped with physical sanitation infrastructure either as a result of infrastructural investments during the socialist era (prior to 1990), because they obtained some funding in early EU projects (e.g., PHARE pre-accession funding, which helped to build water pipelines and sewerage) or due to some recent investment from the state budget or the European Structural and Investment Funds. The land on which Roma dwellings in the settlement areas are built is mostly under the ownership of a village or its individual Roma residents. A municipality will also own some vacant land for possible municipal rental housing construction and physical infrastructure such as water pipelines and sewerage expansion. The mayor of a municipality may or may not be of a Roma ethnic background but local municipal authorities, generally, have some empathy towards the problems that local Roma are facing, and are relatively unbiased and non-discriminatory in their decisions and attitudes towards Roma. This type of village is typically engaged in some developmental “soft” non-investment projects funded by the European Social Fund (most often these are “field social work”, “community work” or similar). Such a municipality, however, might also be involved in “hard” construction projects aimed at the local Roma community, funded by the European Structural and Investment Funds and co-financed by the municipal budget. Although serious problems with regard to housing, fair access to water and sanitation, and multiple health risks are present, there is a sign of some gradual improvement regarding the environmental and health risks, rather than deterioration.

C type: “Not in my backyard” attitude from municipalities. The third type is that of medium or large size villages, which are typically economically better off and located in economically, commercially or touristically attractive regions. The Roma population here live in structurally vulnerable, segregated areas, spatially separated from villages by artificial or natural barriers, e.g., fences, forests or rivers. There often is a lack of political will on behalf of municipality representatives, who typically are non-Roma, to overcome obstacles and enhance the integration and improve the living conditions of Roma. Such a village is in relatively good economical shape. The water pipelines and sewerage are in the possession of the village and dwellings of its non-Roma inhabitants are connected. In contrast, Roma dwellings in a settlement are excluded from municipal development with regard to sanitation infrastructure or new housing. The substantiation of municipal representatives, regarding the differential approach to Roma settlements, is that the “illegal” status (dwellings are non-registered by the Construction Authorities) or bad technical conditions of dwellings prevent the construction of some sanitation system. There is no visible sign of developmental activities or projects for Roma and there is no substantial political will in respect of solving the problems that local Roma face. A village will openly or latently ignore special needs in a Roma settlement and ignores structural inequalities between Roma and non-Roma inhabitants. Village representatives may justify different conditions in which Roma live by means of the “laziness” or “backwardness” of Roma and their alleged preference for living in settlements on the outskirts of villages and “in harmony with nature”. A mayor will be non-Roma and a municipal council will rarely involve a Roma person. Authorities are biased against local Roma, verging on the brink of racism. The “natural order of things” which disempowers and marginalises Roma is internalised, and is further reproduced in local policy and social practice. Such a village may be engaged in some projects funded by the European Social Fund and aimed at Roma. These are mostly “soft” projects such as social work, which is considered to be helpful for officials because project allows funding social workers who take over the “Roma agenda” from the municipal office and, thus, “ease the burden” on administrators having to deal and communicate with Roma. The local and mostly poverty-stricken Roma population often migrate abroad for work to the UK or the Czech Republic, as well as to the core economic region of Slovakia, i.e., the Bratislava region. However, a significant share of the local Roma population remains at home and is dependent on social assistance.

## 4. Discussion: What Works and What Could Work?

The presented typology of governance approaches of municipal authorities towards local Roma leads to a differentiated situation regarding sanitation infrastructure, the prevalence of infectious diseases and in addressing multiple health risks. Identified was a discrepancy between valid laws and norms (on the one hand) and local policies and social practices (on the other hand) regarding the management of multiple health risks in municipalities in areas in which Romani households are situated.

Ascertaining the concept of the Systemic Edge [32], it was assumed that in the gist of the problem causing these disparities is an ongoing move from redistributive economic Keynesianism to neoliberalism of privatisation and economic deregulation, which entails a switch from social dynamics integrating people to dynamics that gravitate towards wider inequalities [33]. This was related also to the fact that governments gradually spend much less on social services than on the needs of corporate economic sectors [15]. In other words, the living conditions of marginalised Roma at the Systemic Edge were deteriorating as a result of these processes, and their chances of a decent life were diminishing.

Generally, the aforementioned theorising might be applied to the situation of the excluded Roma population in settlements and the issue of multiple health risks [38]. However, the fieldwork revealed that there are also examples of good practice and the constructive role of local governance in using opportunities to secure safe environmental and health standards for Roma inhabitants. There was interest from some municipalities in building such sanitation infrastructure, propelled by obligations to comply with EU directives and supported by significant allocations from the European Structural and Investment Funds.

An important aspect of future physical sanitation infrastructure planning should be the consideration of demographic trends in localities and the general developmental potential of towns and villages. A barrier might be the fact that in less populated rural settlements and in localities in which there is widespread poverty and low-income households face difficulty in covering water service costs and fees, infrastructure investments may prove to be unprofitable and economically unreasonable. It has to be taken into consideration that the extremely low income and consumption of households have a direct impact on the health of the population. An unintended unfavourable consequence of investment in physical infrastructure, however, might be the reinforcement of Roma segregation, if infrastructure is constructed in inhabited areas, which are already spatially segregated.

The typology of municipalities in policy towards Roma settlements and addressing multiple health risks indicate that access to safe sanitation was often not so much a problem of knowledge or perception of a situation, but rather interrelated issue of structural conditions and institutional discrimination at the local level [3]. One of the practical good examples that the state authorities should make in this respect was the Portuguese Government’s Strategic Plan (Plano Estratégico de Abastecimento de Água e de Saneamento de Águas Residuais). To comply with the EU directives, that sets clear targets for service coverage to be achieved with the joint contribution of all the authorities involved in water and wastewater services provision, The Strategic Plan defines the strategic objectives and some operational ones, the investments to be made, the management models that could be used to provide the services, the environmental values to be achieved, the financing models and tariff policies, private sector participation, the regulatory model and the legal framework. The Strategic Plan has been very successful in helping to focus the efforts of all stakeholders on priority actions in access to water and sanitation [39]. Thus, there should be a substantial effort made by state authorities to aid municipalities in order to make them overcome structural barriers and secure just and fair treatment for all residents.

Policy priority should be with building sanitation infrastructure in a smart way. This entails goal-oriented planning that analyses costs and benefits based on a comprehensive calculation of the economic returns on investment considering geomorphology, demography and migration, as well as the economic situation of the local population. Furthermore, it appears to be necessary to provide technical assistance to municipalities, benefitting especially small municipalities that do not have the capacity to prepare, fund and implement infrastructure construction projects from the European Structural and Investment Funds [1].

There were obvious limitations of this study that stem from research design and the use of qualitative methods such as ethnographic fieldwork. As limitations can be also seen current operationalisation of the multiple health risks as prevalence of certain contagious diseases and its linear correlation with the absence of sanitation infrastructure. Therefore, it is suggested that, in future, research attention should be focus more on a multifaceted assessment of demographic, geographical, geomorphological factors in rural conditions determining local governmentality. Further research may include combining the calculation of investments costs with health impact assessments (HIAs) [40] and may result in practical alternative solutions for local governments (e.g., building of root water treatment plants) to the existing large-scale, investment-heavy infrastructure projects. Such research might provide an objective and practical data for sanitation and public health local policy planning, utilising already existing epidemiological and social science knowledge as evidence-based decision making.

## 5. Conclusions

This paper attempted to outline modalities of rural municipalities’ attitudes, conduct and responses in managing health risks regarding the local Roma population. It was argued that the effective mitigation of multiple health burdens in Roma settlements was closely related to the presence or absence of sanitation infrastructure. New contributing arguments brought to the scientific discussion in this context were typologies of local governance informed by the mix of structural variables (presence or absence of sanitation infrastructure, demography, presence or absence of development activities) and data from interviews and ethnographic observation. The research results indicated a “triad” of key factors that played a role in local responses to multiple health risks. These were: the structural conditions in municipalities, the history of local inter-ethnic relations and attitudes of authorities towards impoverished Roma. It was also suggested that the problem of lack of access to sanitation cannot be understood without considering social inequalities, institutional discrimination and exclusion dynamics at the Systemic Edge. Such exclusion dynamics were propelled and reinforced by populist politics spreading ethnic stereotypes and reproducing “doxa” (Bourdieu)-society’s taken-for-granted, “unquestioned” truths about Roma who were seen as the undeserving poor and scrounging with respect to welfare.

As the research revealed, although national public health legislation and policies were in place, the approach of village officials to local Roma is often based on “beyond the pale” thinking, and local authorities are often both physically and symbolically excluding segregated settlements from sanitation standards because they do not consider them to be a subject worthy of political attention.

The window of opportunity lies in the EU legislation and funding that should be conducive not only to the enforcement of norms and public health standards, but also to the accumulation of finances for the construction of sanitation infrastructure. This “push” on municipalities, however, has to be complemented by targeted social policies that address low-income households and increase their purchasing power.

Thus, the way out is not only in a compliance with principles of non-discrimination and safeguarding, existing in technical norms and standards, but also in securing accessible funding for infrastructure, which in a smart way, should be innovative including the implementation of cheaper, smaller and environmentally responsible sanitation technologies. Improving the access to sanitation infrastructure, however, requires responsive governance and local strategic planning to safeguard the equality of opportunities for all ethnic and social groups.

## Figures and Tables

**Table 1:** Research grid.

	Structural Variables				Fieldwork Data			
Name of Village	Presence of Ssanitation in the Settlement	Roma Settlement’s Size	Presence of DevelopmentActivities	Disease Incidence (N between Years 2014–2019)	Respondent’s Type Respectively Respondent’s Position	Number of Individual Interviews (N)	Number of Group Discussions (N)	Number of Coded Sections (from Fieldwork Notes) (N)
Chminianske Jakubovany	no	large	no	47	officials, locals	5	1	24
Veľká Lomnica	partially	large	yes	23	officials, locals	5	-	18
Richnava	no	large	no	25	officials, locals	3	1	16
Ostrovany	yes	midsize	yes	11	officials, locals	4	-	15
Kecerovce	partially	large	yes	15	social workers, locals, NGO	5	1	21
Bystrany	no	large	yes	11	officials, social workers, locals	6	2	24
Žehra	partially	midsize	limited	6	social workers, locals, NGO	5	1	12
Smižany	partially	large	yes	13	social workers, locals	4	-	17
Svinia	partially	midsize	yes	35	officials, locals	4	-	14
Jarovnice	partially	large	limited	52	officials, locals	4	1	15
Rakúsy	partially	large	yes	22	officials, locals	3	1	17
Važec	yes	small	no	16	officials	1	-	12
Pečovská Nová Ves	partially	small	limited	10	officials, locals	4	-	10
Letanovce	yes	small	yes	13	social workers, locals, health authority	5	1	14
Rudňany	partially	large	yes	21	officials, locals	4	1	12
Markušovce	partially	large	limited	19	officials, locals	2	-	19
Hanušovce nad Topľou	yes	small	yes	61	officials, locals	3	-	17
Sečovce	partially	midsize	yes	92	social workers, locals	3	1	11
Lomnička	no	large	no	7	officials, locals	3	1	15
Nálepkovo	partially	midsize	yes	6	officials, locals, NGO	4	1	20
Veľké Kapušany	yes	small	yes	11	officials, locals, social workers	3	1	18
Dobšiná	partially	midsize	yes	28	officials, social workers, locals, NGO	6	2	29

## Data Availability

The data presented in this study are available on request from the corresponding author.
